# Supplementary material for: Fetal-restricted hematopoietic progenitors arise from hemogenic endothelium in vitelline and umbilical arteries
Source: Nat Cardiovasc Res. 2026 Apr 8;5(4):308–27. doi: 10.1038/s44161-026-00793-8 (PMC13095662; doi:10.1038/s44161-026-00793-8)
Supplement: Supplementary file 1 — Reporting Summary [file 44161_2026_793_MOESM1_ESM.pdf]

nature portfolio

Corresponding author(s): Emanuele Azzoni

Last updated by author(s): Feb 12, 2026

Reporting Summary

Nature Portfolio wishes to improve the reproducibility of the work that we publish. This form provides structure for consistency and transparency in reporting. For further information on Nature Portfolio policies, see our Editorial Policies and the Editorial Policy Checklist.

Statistics

For all statistical analyses, confirm that the following items are present in the figure legend, table legend, main text, or Methods section.

n/a	Confirmed	
☐	☒	The exact sample size (*n*) for each experimental group/condition, given as a discrete number and unit of measurement
☐	☒	A statement on whether measurements were taken from distinct samples or whether the same sample was measured repeatedly
☐	☒	The statistical test(s) used AND whether they are one- or two-sided<br>*Only common tests should be described solely by name; describe more complex techniques in the Methods section.*
☒	☐	A description of all covariates tested
☐	☒	A description of any assumptions or corrections, such as tests of normality and adjustment for multiple comparisons
☐	☒	A full description of the statistical parameters including central tendency (e.g. means) or other basic estimates (e.g. regression coefficient) AND variation (e.g. standard deviation) or associated estimates of uncertainty (e.g. confidence intervals)
☐	☒	For null hypothesis testing, the test statistic (e.g. *F*, *t*, *r*) with confidence intervals, effect sizes, degrees of freedom and *P* value noted<br>*Give P values as exact values whenever suitable.*
☒	☐	For Bayesian analysis, information on the choice of priors and Markov chain Monte Carlo settings
☒	☐	For hierarchical and complex designs, identification of the appropriate level for tests and full reporting of outcomes
☒	☐	Estimates of effect sizes (e.g. Cohen's *d*, Pearson's *r*), indicating how they were calculated

Our web collection on statistics for biologists contains articles on many of the points above.

Software and code

Policy information about availability of computer code

Data collection

Imaging Data:
Zeiss Zen software (Zen Black) version 2.3 SP1 FP3
Fiji/ImageJ Cell Counter tool (v.2.3.5-2.9.0)
Imaris (v 9.7.2)

Flow cytometry Data:
BD FACSDiva software (version 8.0.2)
Summit software version 6.3
BD FACS Chorus software 6.1.0.

Data analysis

Imaging Data analysis:
Imaris Viewer (v.10.2)
Imaris (v. 9.7.2)
Fiji/ImageJ (v.2.3.5-2.9.0)
Adobe Photoshop 2024

Flow Cytometry Data Analysis:
FlowJo (v. 10)

scRNA-Seq Data analysis:

Seurat (v. 4.0)
SCTransform
R (R-3.2.3 – R-4.2.1)
Louvain
CellRanger (v 6.1)
Clustree (v. 0.4.3)
SingleR (v1.0.1)
ShinyCell (https://github.com/SGDDNB/ShinyCell)
ClusterProfiler (v. 4.8.3)
glmGamPoi (https://github.com/const-ae/glmGamPoi)

Statistics:
GraphPad Prism (v. 10.2.1-10.6.1)
Microsoft Excel (v. 16.96 and earlier versions)

For manuscripts utilizing custom algorithms or software that are central to the research but not yet described in published literature, software must be made available to editors and reviewers. We strongly encourage code deposition in a community repository (e.g. GitHub). See the Nature Portfolio guidelines for submitting code & software for further information.

Data

Policy information about availability of data

All manuscripts must include a data availability statement. This statement should provide the following information, where applicable:

- Accession codes, unique identifiers, or web links for publicly available datasets
- A description of any restrictions on data availability
- For clinical datasets or third party data, please ensure that the statement adheres to our policy

Raw scRNA-seq data of YS+VU and AGM from E10.5 Cdh5-CreERT2::R26tdTomato embryos (4-OHT at E8.5) are available at the NCBI Sequence Read Archive (SRA) data repository with the accession number BioProject ID: PRJNA898269 (sample accession numbers: SRR28006358, SRR28006359, SRR28006360, SRR28006361).

The following published datasets were used in this study:
GSE180357 (Patel et al., 2022)
GSE143637 (Vink et al., 2020)

Research involving human participants, their data, or biological material

Policy information about studies with human participants or human data. See also policy information about sex, gender (identity/presentation), and sexual orientation and race, ethnicity and racism.

Reporting on sex and gender	N/A
Reporting on race, ethnicity, or other socially relevant groupings	N/A
Population characteristics	N/A
Recruitment	N/A
Ethics oversight	N/A

Note that full information on the approval of the study protocol must also be provided in the manuscript.

Field-specific reporting

Please select the one below that is the best fit for your research. If you are not sure, read the appropriate sections before making your selection.

☒ Life sciences ☐ Behavioural & social sciences ☐ Ecological, evolutionary & environmental sciences

For a reference copy of the document with all sections, see nature.com/documents/nr-reporting-summary-flat.pdf

Life sciences study design

All studies must disclose on these points even when the disclosure is negative.

Sample size	For in vivo experiments, whenever possible the number of samples required to obtain statistically reliable data, while avoiding both an excess of animals used for each experiment and unnecessary repetitions, was calculated using the G*Power package (version 3.1.9.3) based on data in the literature, conducted using similar models. The number of samples for each group was set to highlight an effect size of 1.2 (with a power of at least 80%, error α = 0.05). In the remaining cases no statistical method was used to pre-determine sample size, but sample sizes are consistent with our previous studies and other studies in the field.

Data exclusions

No data were excluded from flow cytometry, imaging and CFU-C experiments.
For OP9 co-cultures, one experiment was excluded from final analysis due to a very low (near zero) initial percentage of Kit+ progenitors labelling, that was interpreted as inefficient 4OHT-dependent recombination, likely due to technical problems with I/P injection in one single mouse.
In E11.5 AGM/YS-VU transplantation experiments 4 mice from 4-OHT E8.5 AGM and 7 mice from 4-OHT E8.5 YS-VU were excluded from analysis, because the chimerism was under the predetermined treshold of 5% from the first PB analysis.
In E14.5 FL transplantation experiments (secondary transplant), one mouse from the 4OHT E8.5-eYFP group, and two mice from the 4OHT E10.5-tdTomato group were excluded from the analysis as they died between 3 weeks and 1 month post-transplant. The most likely cause cause of death in these mice was fight wounds.
For scRNA-Seq, cells with genes count < 300 and > 8000 and fraction of mitochondrial reads > 0.20 were excluded from downstream processing.

Replication

Numbers of biological replicates and independent experiments (different litters) are indicated in figure legends and each experiment was replicated at least once (N=2 independent biological replicates)
For flow cytometry and imaging experiments, embryos were analyzed individually and each individual embryo is considered as one biological replicate. For ex vivo CFU-C assays and bulk OP9 co-cultures, each biological replicate was further split into two technical replicates, then averaged in the analysis. For ex vivo LDA experiments several e.e. from a litter were pooled together (7 to 12 e.e.), and sorted cells were seeded at the doses of 10, 30, 100 or 200 cells per well, in a minimum of 8 and a maximum of 12 wells per condition. Each pool is considered a biological replicate. For E11.5 AGM/YS-VU transplantation experiments 2 e.e. were transplanted in each recipient, considered as a biological replicate. For E14.5 FL transplantation experiments, each recipient mouse was transplanted with cells from a different embryo and considered a biological replicate.

Randomization

No specific randomization method was used. Animals were allocated into experimental groups according to their genotype.

Blinding

No specific methods were used for blinding. The investigators were not blinded to group allocations during data collection and analysis.

Reporting for specific materials, systems and methods

We require information from authors about some types of materials, experimental systems and methods used in many studies. Here, indicate whether each material, system or method listed is relevant to your study. If you are not sure if a list item applies to your research, read the appropriate section before selecting a response.

Materials & experimental systems

n/a	Involved in the study
☐	☒ Antibodies
☐	☒ Eukaryotic cell lines
☒	☐ Palaeontology and archaeology
☐	☒ Animals and other organisms
☒	☐ Clinical data
☒	☐ Dual use research of concern
☒	☐ Plants

Methods

n/a	Involved in the study
☒	☐ ChIP-seq
☐	☒ Flow cytometry
☒	☐ MRI-based neuroimaging

Antibodies

Antibodies used

Antibodies used in this study are listed below (antibody name; clone name where applicable; Supplier name; Cat. number; RRID identifier; working dilution)
FLOW CYTOMETRY ANTIBODIES
Rat monoclonal anti-Ter119 APC-fire750 (clone TER-119) BioLegend Cat#116250; RRID: AB_2819833 1:200
Rat monoclonal anti-CD117 (c-Kit) FITC (clone 2B8) eBioscience Ref: 11-1171-85; RRID: AB_465187 1:100
Rat monoclonal anti-CD41 PE-Cy7 (clone eBioMWReg30) eBioscience Ref: 25-0411-82; RRID: AB_1234970 1:200
Rat monoclonal anti-CD16/32 APC (clone 93) BioLegend Cat#101326; RRID: AB_1953273 1:200
Rat monoclonal anti-CD150 PE-Cy7 (SLAM) (clone TC15-12F12.2) BioLegend Cat#115914; RRID: AB_439797 1:400
Armenian hamster monoclonal anti-CD48 APC (clone HM48-1) BioLegend Cat#103412; RRID: AB_571997 1:400
Rat monoclonal anti-Ly6A/E (Sca-1) Pacific Blue (clone E13-161.7) BioLegend Cat#122520; RRID: AB_2143237 1:100
Rat monoclonal anti-CD117 (c-kit) APC-Cy7 (clone 2B8) BioLegend Cat#105826; RRID: AB_1626278 1:600
Rat monoclonal anti-CD45 APC-eFluor 780 (clone 30-F11) eBioscience Cat#47-0451-82; RRID: AB_1548781 1:200
Rat monoclonal anti-CD117 (c-kit) PE-Cy7 (clone 2B8) BioLegend Cat#105814; RRID: AB_313223 1:100
Rat monoclonal anti-CD135 Brilliant Violet 421 (clone A2F10) BioLegend Cat#135313; RRID: AB_2562338 1:200
Rat monoclonal anti-CD127 (IL-7Ra) PE (clone A7R34) BioLegend Cat#135009; RRID: AB_1937252 1:200
Rat monoclonal anti-Ter119 PE-Cy5 (clone TER-119) BioLegend Cat#116210; RRID: AB_313711 1:400
Armenian hamster monoclonal anti-CD3e PE-Cy5 (clone 145-2C11) BioLegend Cat#100310; RRID: AB_312675 1:200
Rat monoclonal anti-F4/80 PE-Cy5 (clone BM8) BioLegend Cat#123112; RRID: AB_893482 1:200
Mouse monoclonal anti-NK1.1 PE-Cy5 (clone PK136) BioLegend Cat#108716; RRID: AB_493590 1:400
Rat monoclonal anti-Ly6G/Ly6C (Gr1) PE-Cy5 (clone RB6-8C5) BioLegend Cat#108410; RRID: AB_313375 1:400
Rat monoclonal anti-CD19 PE-Cy5 (clone 6D5) BioLegend Cat#115509; RRID: AB_313644 1:400
Rat monoclonal anti-CD45R/B220 PE-Cy5 (clone RA3-6B2) BioLegend Cat#103210; RRID: AB_312995 1:200
Rat monoclonal anti-CD45 PE (clone 30-F11) BioLegend Cat#103106; RRID: AB_312971 1:200
Rat monoclonal anti-CD45R/B220 APC (clone RA3-6B2) BioLegend Cat#103212; RRID: AB_312997 1:200

Rat monoclonal anti-CD45R/B220 APC-Cy7 (clone RA3-6B2) BioLegend Cat#103224; RRID: AB_313007 1:200
Rat monoclonal anti-CD11b PE-Cy7 (clone M1/70) BioLegend Cat#101216; RRID: AB_312799 1:200
Rat monoclonal anti-Ly6G/Ly6C (Gr1) APC (clone RB6-8C5) BioLegend Cat#108412; RRID: AB_313377 1:200
Mouse monoclonal anti-CD45.2 FITC (clone 104) BioLegend Cat#109806; RRID: AB_313443 1:400
Mouse monoclonal anti-CD45.1 PE (clone A20) BioLegend Cat#110708; RRID: AB_313497 1:200
Mouse monoclonal anti-CD45.1 BV786 (clone A20) BioLegend Cat#110743; RRID: AB_2563379 1:200
Rat monoclonal anti-CD93 (AA4.1) APC (clone AA4.1) BioLegend Cat#136510; RRID: AB_2275868 1:200
Rat monoclonal anti-F4/80 APC (clone BM8) BioLegend Cat#123116; RRID: AB_893481 1:200
Rat monoclonal anti-CD4 PE-Cy5 (clone RM4-5) BioLegend Cat#100514; RRID: AB_312717 1:200
Rat monoclonal anti-CD8a APC-Cy7 (clone 53-6.7) BioLegend Cat#100714; RRID: AB_312753 1:200
Rat monoclonal anti-CD25 PE-Cy7 (clone PC61) BioLegend Cat#102016; RRID: AB_312865 1:200
Rat monoclonal anti-CD44 APC (clone IM7) BioLegend Cat#103012; RRID: AB_312963 1:400
Mouse monoclonal anti-CD45.2 APC-Cy7 (clone 104) BioLegend Cat#109824; RRID: AB_830789 1:100
Rat monoclonal anti-CD31 APC (clone 390) BioLegend Cat#102410; RRID: AB_312905 1:200
Rat monoclonal anti-CD117 (c-kit) BV 786 (clone 2B8) BD Horizon Cat#564012; RRID: AB_2732005 1:200
Rat monoclonal anti-CD117 (c-kit) APC (clone 2B8) BioLegend Cat#105812; RRID: AB_313221 1:200
Rat monoclonal anti-CD117 (c-kit) PE (clone 2B8) BioLegend Cat#105807; RRID: AB_313216 1:600
Rat monoclonal anti-CD201 PE (clone 1560) BD Pharmingen Cat#566337; AB_2739694 1:100
Rat monoclonal anti-mouse CD16/CD32 antibody (Fc Block), clone 2.4G2 BD Biosciences Cat# 553142; RRID:AB_394657 1:500
Rat monoclonal Anti-Mouse CD44 BV510 (clone IM7) BD Biosciences Cat#563114; RRID: AB_2738011 1:400
Armenian Hamster monoclonal Anti-Mouse CD3e BUV395 (clone 145-2C11) BD Biosciences Cat#563565; RRID: AB_2738278 1:100
Rat monoclonal Anti-Mouse CD4 BV786 (clone RM4-5) BD Biosciences Cat#563727; RRID: AB_2728707 1:400
Rat monoclonal Anti-Mouse CD8a Pacific Blue (clone 53-6.7) BioLegend Cat#100725; RRID: AB_493425 1:300
Rat monoclonal Anti-Mouse CD43 Alexa Fluor 700 (clone S11) BioLegend Cat#143213; RRID: AB_2800660 1:200
Human recombinant monoclonal Anti-Mouse CD45 APC Vio770 (clone REA737) Miltenyi Cat#130-110-662; RRID: AB_2658231 1:200
Rat monoclonal Anti-Mouse CD25 APC (clone PC61) BD Biosciences Cat# 557192; RRID:AB_398623 1:200
Rat monoclonal Anti-Mouse CD4 Pacific Blue (clone RM4-5) BD Biosciences Cat# 558107; RRID:AB_397030 1:200
Rat monoclonal Anti-Mouse CD8a PE (clone 53-6.7) BD Biosciences Cat# 553033; RRID:AB_394571 1:200
Rat monoclonal Anti-Mouse CD45 BV605 (clone 30-F11) BioLegend Cat# 103139; RRID: AB_2562341 1:200
Rat monoclonal Anti-Mouse CD11b FITC (clone M1/70) BioLegend Cat#101206; RRID: AB_312789 1:200
Rat monoclonal Anti-Mouse F4/80 AF700 (clone BM8) BioLegend Cat#123130; RRID: AB_2293450 1:200
Rat monoclonal Anti-Mouse CD4 BV786 (clone RM4-5) eBioscience Cat# 417-0042-82; RRID: AB_2921053 1:200
Rat monoclonal Anti-Mouse CD8a PE-Cy7 (clone 53-6.7) eBioscience Cat# 25-0081-82; RRID: AB_469584 1:200
Rat monoclonal Anti-Mouse CD45R/B220 APC (clone RA3-6B2) BD Biosciences Cat#553092; RRID: AB_398531 1:200
Rat monoclonal Anti-Mouse F4/80 FITC (clone BM8) BioLegend Cat#123108; RRID: AB_893502 1:200
Rat monoclonal Anti-Mouse Ter119 APC-ef780 (clone TER119) eBioscience Cat# 47-5921-80; RRID: AB_1548786 1:200
Streptavidin BV785 BioLegend Cat#405233 1:200
Rat monoclonal Anti-Mouse Sca1 FITC (clone E13-161.7) BioLegend Cat# 122506; RRID: AB_756191 1:200
Rat monoclonal Anti-Mouse CD150 PE-Cy7 (clone mShad150) eBioscience Cat# 25-1502-82 RRID: AB_10805742 1:200
Armenian Hamster monoclonal Anti-Mouse CD48 APC-Cy7 (clone HM48-1) BD Biosciences Cat# 561242; RRID: AB_10644381 1:100
Rat monoclonal Anti-Mouse CD45R/B220 Biotin (clone RA3-6B2) BD Biosciences Cat# 553086; RRID: AB_394615 1:200
Armenian Hamster monoclonal Anti-Mouse CD3e Biotin (clone 145-2C11) BD Biosciences Cat#553060; RRID: AB_394593 1:200
Rat monoclonal Anti-Mouse F4/80 Biotin (clone BM8) eBioscience Cat# 13-4801-85; RRID: AB_466657 1:200
Rat monoclonal Anti-Mouse Gr-1 Biotin (clone RB6-8C5) BD Biosciences Cat# 553125; RRID: AB_394641 1:200
Mouse monoclonal Anti-Mouse Nk1.1 Biotin (clone PK136) Biolegend Cat# 108704; RRID: AB_313391 1:200
Rat monoclonal Anti-Mouse Csf1r Biotin (clone AFS98) BioLegend Cat# 135508; RRID: AB_2085223 1:200
Rat monoclonal Anti-Mouse CD4 Biotin (clone GK1.5) BD Biosciences Cat# 553728; RRID: AB_395012 1:200
Rat monoclonal Anti-Mouse CD8a Biotin (clone 53-6.7) eBioscience Cat# 13-0081-85; RRID: AB_466346 1:200
Armenian Hamster monoclonal CD11c Biotin (clone N418) BioLegend Cat# 117304; RRID: AB_313773 1:200
Rat monoclonal Anti-Mouse Ter119 Biotin (clone TER119) BD Biosciences Cat# 553672; RRID: AB_394985 1:200

IMMUNOFLUORESCENCE ANTIBODIES
Goat polyclonal anti-m/rCD31/PECAM1 R&D systems Cat#AF3628 1:100
Rabbit polyclonal anti-GFP Invitrogen Cat#A11122; RRID: AB_221569 1:300
Chicken polyclonal anti-GFP Invitrogen Cat#A10262; RRID: AB_2534023 1:500
Rat monoclonal anti-mouse CD117 (c-Kit) (clone 2B8) eBioscience Cat# 14-1171-82; RRID: AB_467433 1:200
Rabbit polyclonal anti-RFP Rockland Cat# 600-401-379; 1:500
Armenian hamster monoclonal anti-mouse CD27 (clone LG-7F9) eBioscience Cat#14-0271-82; RRID: AB_467183 1:100
Rabbit anti-mouse/rat/human Runx1, clone EPR3099 Abcam Cat# ab92336; RRID: AB_2049267 1:400
Donkey polyclonal anti-goat Alexa Fluor Plus 647 Invitrogen Cat#A32849; RRID: AB_2762840 1:500
Donkey polyclonal anti-rabbit Alexa Fluor Plus 488 Invitrogen Cat#A32790; RRID: AB_2762833 1:500
Donkey polyclonal anti-rabbit Alexa Fluor Plus 555 Invitrogen Cat#A32794; RRID: AB_2762834 1:500
Donkey polyclonal anti-rat Alexa Fluor 488 Invitrogen Cat#21208; RRID: AB_2535794 1:500
Donkey polyclonal anti-rat CF568 Biotium Cat#20092; RRID: AB_ 1:500
Donkey anti-chicken IGY AF488 Invitrogen Cat#A78948; RRID: AB_2921070 1:500
Goat polyclonal anti-armenian hamster DyLight 649 (clone Poly4055) BioLegend Cat#405505; RRID: AB_1575122 1:500

Validation

All antibodies have been validated by the manufacturer for the respective specific applications, and each lot has been tested for performance.

Eukaryotic cell lines

Policy information about cell lines and Sex and Gender in Research

Cell line source(s)

OP9: ATCC CRL-2749. OP9-Dl1 were generated in the lab of Juan Carlos Zúniga-Pflücker.

Authentication	No new cell lines were generated in this study. No lines were authenticated.
Mycoplasma contamination	OP9 and OP9-Dl1 cells tested negative for mycoplasma contamination during routine checks.
Commonly misidentified lines (See ICLAC register)	No commonly misidentified lines in the ICLAC registry were used for the present study.

Animals and other research organisms

Policy information about studies involving animals; ARRIVE guidelines recommended for reporting animal research, and Sex and Gender in Research

Laboratory animals	The transgenic mouse lines used in the present study were the following (indicated are name of the mouse line, source and RRID and/or reference).<br>Cdh5-CreERT2 - Gift from R.Adams (Wang et al., 2010)<br>R26zsGreen - Gift from M.Iannacone - RRID:IMSR_JAX:007906<br>R26tdTomato - The Jackson Laboratory - RRID:IMSR_JAX:007909<br>R26EYFP - The Jackson Laboratory - RRID:IMSR_JAX:006148<br>Csf1r-iCre - (Deng et al., 2010) - RRID:IMSR_JAX:021024<br>Csf1rMerCreMer (Qian et al, 2011) RRID:IMSR_JAX:019098<br>B6.SJL-Ptprca Pepcb/BoyJ (B6 CD45.1) - Gift from L. Naldini - RRID:IMSR_JAX:002014<br><br>All transgenic mouse lines were maintained on a CD45.2 C57BL/6 genetic background, with the exception of females used for timed matings in order to generate adult mice with 4-OHT activation during embryogenesis, which were instead of C57BL/6/FVB mixed background (F1). Mouse age is indicated in the manuscript and was comprised between 3 and 12 weeks at the time of the start of the experiment. Mice were housed in individually ventilated cages or filter top cages with a 12h:12h light-dark cycle (350/450 lux) and unrestricted access to food and water in the animal facilities at the San Raffaele Scientific Institute, University of Oxford or at the University of Milan. Standardized housing conditions included 22 (+/-2) °C temperature and relative humidity of 55% (+/-5%).
Wild animals	This study does not involve wild animals.
Reporting on sex	Sex information was not considered in the study design, and was not collected in embryos analyzed in this study. No sex-based difference was observed in analysis of adult mice. Hence, findings of the present study apply to both sexes.
Field-collected samples	This study does not involve field collected samples.
Ethics oversight	All mouse experiments were performed in accordance with experimental protocols approved by San Raffaele Scientific Institute and University of Milan Institutional Animal Care and Use Committees (IACUC) and authorized by the Italian Ministry of Health (Authorization numbers n° 503/2019-PR, 753/2023-PR and 351/2022-PR). All procedures carried out at the University of Oxford were in compliance with United Kingdom Home Office regulations and the Oxford University Clinical Medicine Animal Welfare and Ethical Review Committee (PPL number: PP9552402).

Note that full information on the approval of the study protocol must also be provided in the manuscript.

Plants

Seed stocks	N/A
Novel plant genotypes	N/A
Authentication	N/A

Flow Cytometry

Plots

Confirm that:

☒ The axis labels state the marker and fluorochrome used (e.g. CD4-FITC).

☒ The axis scales are clearly visible. Include numbers along axes only for bottom left plot of group (a 'group' is an analysis of identical markers).

☒ All plots are contour plots with outliers or pseudocolor plots.

☒ A numerical value for number of cells or percentage (with statistics) is provided.

Methodology

Sample preparation	Sample preparation is detailed in the Methods section. Briefly, single cell suspensions were obtained from embryonic tissues (yolk sac, embryo/caudal part, thymus, fetal liver) by incubating for 20min at 37°C in calcium/magnesium free PBS supplemented with FBS 10%, 50 U/ml penicillin, 50 mg/ml streptomycin, , EDTA 2mM and collagenase type I (Sigma) 0.12% (w/v), followed by mechanical dissociation by pipetting. Peripheral blood (PB) samples were collected by tail vein bleeding using a scalpel; bone marrow (BM) was obtained by flushing long bones using a syringe and filtered in 40 micron strainers. PB, BM and fetal liver samples were treated with the appropriate amount of RBC Lysis Buffer. For flow cytometry analysis and cell sorting, single cell suspensions were incubated with conjugated antibodies at 4°C in the dark for 15 minutes.
Instrument	Flow cytometry data acquisition was performed on a BD LSR Fortessa X-20. Cell sorting was performed using a MoFLO Astrios cell sorter (Beckman Coulter), BD FACSAria II or BD FACSDiscover S8
Software	Flow cytometry data was collected with either BD FACSDiva software (version 8.0.2), Summit version 6.3 (Beckman Coulter) or Chorus version 6.1.0 (BD). Flow cytometry data was analyzed using FlowJo software version 10 (BD).
Cell population abundance	Cell populations in bulk were sorted to >95% purity, determined by re-analysis of sorted fractions.
Gating strategy	Cells were identified by FSC-A and SSC-A parameters, followed by doublet exclusion based on the ratio of FSC-W vs FSC-H and SSC-A vs SSC-W. Dead cells were subsequently excluded based on Hoechst 33258 or 7-AAD incorporation. A representative gating strategy for all experiments is included in main and/or Extended Data figures.<br><br>Fig.1B: Cells were gated on Ter119-<br>Fig. 1E: Cells were gated on Lin- (B220, CD19, CD3e, F4/80, Gr1, Nk1.1, Ter119)<br>Fig. 5B: Cells were gated on singlets<br>Extended data Fig. 1B: Cells were gated on Ter119-<br>Extended data Fig. 1F: Cells were gated on CD45+<br>Extended data Fig.2A: Cells were gated on Ter119-<br>Extended data Fig.2C: Cells were gated on Lin- (B220, CD19, CD3e, F4/80, Gr1, Nk1.1, Ter119)<br>Extended data Fig. 3A:Cells were gated on Live (Hoechst-)<br>Extended data Fig. 3B: Cells were gated on Live (Hoechst-)<br>Extended data Fig 3D: Cells were gated on Live (Hoechst-)<br>Extended data Fig. 4C: Cells were gated on Ter119-<br>Extended data Fig. 5C: Cells were gated on Ter119-<br>Extended data Fig 6A: Cells were gated on CD45+<br>Extended data Fig. 6C: Cells were gated on Live (Hoechst-)<br>Extended data Fig.6G: Cells were gated on singlets<br>Extended data Fig.6H: Cells were gated on singlets<br>Extended data Fig. 10A and B: Cells were gated on Live (Hoechst-)<br>Extended data Fig. 10E and G: Cells were gated on Live (Hoechst-)

☒ Tick this box to confirm that a figure exemplifying the gating strategy is provided in the Supplementary Information.
